# Risk factors for post-traumatic stress disorder in acute trauma patients: A protocol for systematic review and meta-analysis

**DOI:** 10.1097/MD.0000000000025616

**Published:** 2021-04-30

**Authors:** Furong Tang, Jianghong Tan, Xi Guo, Jinlian Huang, Jinhua Yi, Lang Wang

**Affiliations:** a Nursing Department; b Emergency Department; c Department of Metabolic Endocrinology, Zhuzhou Central Hospital, Zhuzhou, Hunan province, China.

**Keywords:** acute trauma, meta-analysis, post-traumatic stress disorder, protocol, risk factors

## Abstract

**Background:**

Post-traumatic stress disorder (PTSD) is one of the most commonly reported mental health consequences, followed by disasters and traumatic events, either natural or man-made. At present, there are no unified results for the prevalence rate of PTSD in patients suffering from acute trauma and related influencing factors. Therefore, the purpose of this study is to systematically evaluate the existing literatures, thus obtaining a comprehensive estimation of the combined prevalence rate of PTSD and related factors in trauma patients, so as to provide evidence support for clinical disease prediction models and intervention strategies.

**Methods:**

Published articles will be retrieved from PubMed, Embase, Cochrane Library, Web of Science, China Biology Medicine Database, China National Knowledge Infrastructure, China Science and Technology Journal Database, and Wanfang Database. Research reports will be searched in March 2021. STATA 14.0 software will be applied for data analysis. Mantel–Haenszel fixed effect model or DerSimonian–Laird random effect model will be selected to estimate the pooled prevalence of PTSD in patients with acute trauma and associated factors.

**Results:**

We will disseminate the findings of this systematic review and meta-analysis via publications in peer-reviewed journals.

**Conclusions:**

The results of this analysis can be used to establish a risk prediction model of PTSD in patients experiencing acute trauma, so as to provide intervention strategies.

**OSF Registration Number:**

DOI 10.17605/OSF.IO/Z275U.

## 1 Introduction

Post-traumatic stress disorder (PTSD) is a delayed and persistent mental disorder caused by unusual threatening or catastrophic psychological trauma.^[1–3]^ The main manifestations include repeated intrusive traumatic experience, continuous high vigilance, and avoidance.^[4–6]^ PTSD has been confirmed by a large number of survivors of catastrophic accidents, wars and other traumatic events, with the incidence of 10%, 20%, and 7.8% in the general population.^[7]^

In recent years, with the rapid development of society, transportation, industry, sports, and agriculture have led to an increasing incidence of injuries.^[8]^ Severe trauma can induce systemic reaction, with local manifestations of swelling, tenderness and pain, deformity and dysfunction in the case of fracture and dislocation, and fatal asphyxia, disturbance of consciousness and shock in severe cases. As a common post-traumatic complication, PTSD has serious impacts on the recovery of patients’ social function. Related studies have revealed that, by improving clinicians’ understanding of PTSD, early screening and formulating effective, scientific and correct intervention measures, and clarifying relevant pathogeneses of PTSD, it is of great significance to diagnose and treat PTSD, so as to recover patients’ social function.^[9]^

In recent years, more and more studies on PTSD patients with acute trauma abroad have proved that patients with acute trauma are the victims of PTSD.^[10,11]^ At present, no effective treatment for PTSD is found. About 50% of patients are delayed to the chronic course of disease that in patients with PTSD is more than 10 years, which seriously affects the patients’ life quality.^[7]^ It is very important to prevent the occurrence of PTSD. The risk factors of PTSD include demography, disease severity, psychosocial factors, and so on. However, the current research results are not consistent.

In order to better understand the impacts of PTSD on patients with acute trauma, and to guide clinical practices and future researches, we conducted a systematic review and meta-analysis. The specific purpose of this systematic review and meta-analysis is to quantitatively summarize the prevalence of PTSD in patients with acute trauma. On the other hand, we try to investigate the potential predictors and related factors of these diseases.

## 2 Methods

### 2.1 Study registration

This protocol has been registered on Open Science Framework (grant number: DOI 10.17605/OSF.IO/Z275U). This report will be based on the preferred reporting items for systematic review and meta-analysis protocols.^[12]^

### 2.2 Identification and selection of studies

A systematic review of published research that reports the prevalence and associated factors of PTSD in acute trauma patients will be considered. A search of research reports will be made by the following databases: PubMed, Embase, Cochrane Library, Web of Science, China Biology Medicine Database, China National Knowledge Infrastructure, China Science and Technology Journal Database, and Wanfang Database. Research reports in March 2021 will be included. These search terms are summarized in Table 1.

**Table 1:** Search strategy of the PubMed.

Number	Search terms
#1	Stress disorders, post-traumatic[MeSH]
#2	Neuroses, post-traumatic[Title/Abstract]
#3	PTSD[Title/Abstract]
#4	Post-traumatic stress disorders[Title/Abstract]
#5	Acute post-traumatic stress disorder[Title/Abstract]
#6	Chronic post-traumatic stress disorder[Title/Abstract]
#7	Delayed onset post-traumatic stress disorder[Title/Abstract]
#8	Neuroses, post-traumatic[Title/Abstract]
#9	Posttraumatic stress disorders[Title/Abstract]
#10	Stress disorder, post-traumatic[Title/Abstract]
#11	Stress disorders, post-traumatic[Title/Abstract]
#12	Acute post traumatic Stress disorder[Title/Abstract]
#13	Chronic post traumatic stress disorder[Title/Abstract]
#14	Delayed onset post traumatic stress disorder[Title/Abstract]
#15	Neuroses, post praumatic[Title/Abstract]
#16	Post traumatic stress disorders[Title/Abstract]
#17	Post-traumatic neuroses[Title/Abstract]
#18	Post-traumatic stress disorder[Title/Abstract]
#19	Post-traumatic neuroses[Title/Abstract]
#20	Post-traumatic stress disorder[Title/Abstract]
#21	Stress disorder, post-traumatic[Title/Abstract]
#22	stress disorder, Post-traumatic[Title/Abstract]
#23	Stress disorders, post traumatic[Title/Abstract]
#24	or/1–23
#25	Trauma[Title/Abstract]
#26	Risk factor[Title/Abstract]
#27	Risk assessment[Title/Abstract]
#28	Multivariate analysis[Title/Abstract]
#29	Multivariable logistic regression[Title/Abstract]
#30	or/26–29
#31	#24 and #25 and #30

### 2.3 Eligibility criteria

#### 2.3.1 Inclusion criteria

All relevant research reports that will be available on the search until March 2021 will be included based on the following inclusion criteria:

1. The subjects were patients with acute trauma, aged over 18 years;
2. The contents of the study are the risk factors or predictive factors of PTSD;
3. Using one or more PTSD symptom assessment tools or PTSD diagnostic criteria;
4. Case-control study, cohort study and cross-sectional study;
5. The results of the study involve the specific values of odds ratio and 95% confidence interval of risk factors.

#### 2.3.2 Exclusion criteria

1. Experimental, qualitative, and psychometric studies;
2. Repeatedly published literatures;
3. The full text cannot be obtained normally or the extracted data are affected.

### 2.4 Data extraction

**Figure 1:**
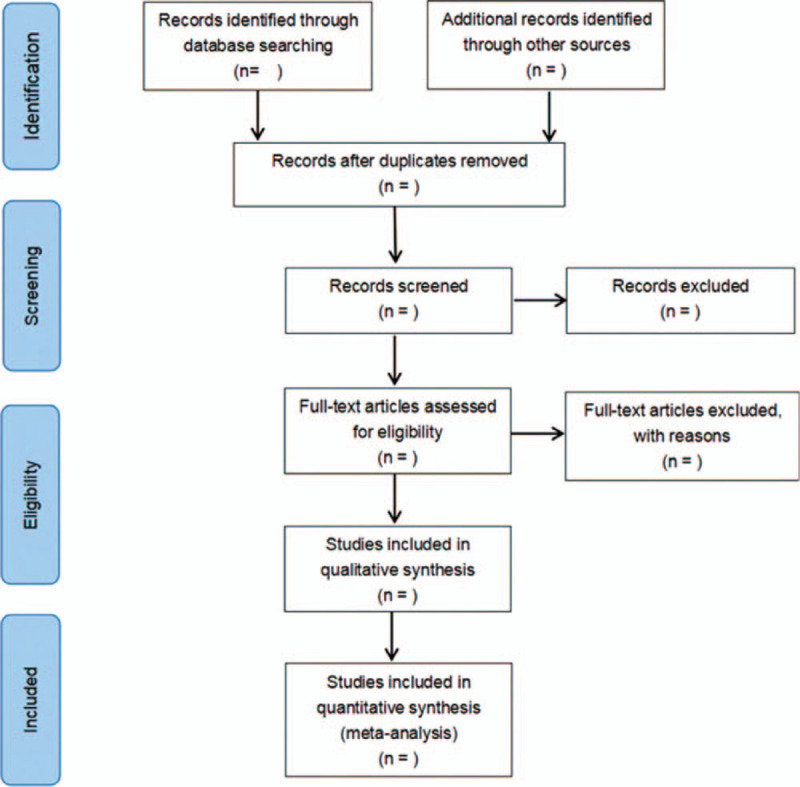
PRISMA flow diagram of the study selection process.

The process of the selection is exhibited in Figure 1. All the literatures are based on Endnote X7 software. First of all, the title and abstract were screened independently by 2 researchers. All the articles of primary screening were read and screened again after reading the full text. If there is any objection to the literature, a consensus can be reached through consultation with a third party. The extracted data are as follows: the first author, publication year, region of the study conducted, sample size, study type, characteristics of people who lost follow-up, risk factors involved, follow-up methods, PTSD evaluation time, evaluation tools, and criteria. If necessary, contacting the original author to obtain further information about the research.

### 2.5 Outcome measurements

We have 2 goals in this systematic review and meta-analysis: to determine the common prevalence of PTSD in patients with acute trauma and to estimate the related factors of PTSD in patients with acute trauma. The combined prevalence of PTSD will be calculated using STATA 14.0. The estimated value of the combined effects of the factors related to PTSD will be calculated. The odds ratio will be prepared from the searched research reports using 2 tables.

### 2.6 Quality assessment

The quality of the research reports included will be assessed using the Newcastle–Ottawa Scale for quality assessment.^[13]^ Newcastle–Ottawa Scale score ≥6 means that the literature quality is better.^[14]^

### 2.7 Data analysis

The data analysis will be conducted through STATA 14.0, and we will use OR and 95%CI to represent. Mantel–Haenszel fixed effect model or DerSimonian–Laird random effect model will be adopted to estimate the pooled prevalence of PTSD in patients with acute trauma and associated factors. If there are no findings of statistical heterogeneity, the Mantel–Haenszel fixed effect model is adopted for data synthesis.^[15]^ If there is significant statistical heterogeneity, we will apply the DerSimonian–Laird random effect model.^[16]^

### 2.8 Assessment of heterogeneity

The prevalence of the reported researches will be checked for heterogeneity by performing a heterogeneity *χ*^*2*^ test and *I*^*2*^ test. When *P* < .1 and/or *I*^*2*^ > 50%, the random effect model is adopted for the combined analysis. Otherwise, the fixed effect model is used for the combined analysis.

### 2.9 Additional analysis

#### 2.9.1 Subgroup analysis

According to trauma type, gender, mean age, study type, study quality, and time between trauma occurrences, we make a subgroup analysis

#### 2.9.2 Meta-regression analyses

We conducted meta-regression analyses to explore source of heterogeneity on a range of study characteristics (trauma type, gender, age, study type, study quality, and time between trauma occurrences). We first conducted univariate meta-regression, followed by multivariable analysis including factors that reached statistical significance in the univariate models.

#### 2.9.3 Sensitivity analysis

To determine the stability of the outcome measures, each outcome measure was analyzed by performing sensitivity analysis.

#### 2.9.4 Assessment of reporting biases

We will evaluate the possibility of publication bias using funnel plots and Egger test of bias will be taking as a complement.^[17]^

### 2.10 Management of missing data

We will try our best to ensure the integrity of the data. If the included data is not complete, we will take every effort to contact the corresponding author of the article, including sending emails or making a phone call. If the corresponding author cannot be contacted, we will remove the experiment with incomplete data. After data integrity is assured, intention analysis therapy and sensitivity analysis will be carried out.

### 2.11 Ethical review and informed consent of patients

The content of this article does not involve moral approval or ethical review and will be presented in print or at relevant conferences.

## 3 Discussion

The manifestations of PTSD are various, including the separation of symptoms, repeated repetition of injury events, deliberate avoidance and so on.^[18–20]^ Among them, the symptoms of separation mainly include insufficient consciousness, slow response, and split amnesia. The repeated re-experience of injury events refers to the refeeling of traumatic events and related people and objects. The improvement of alertness is mainly manifested by anxiety, sleep disorders, irritability, and increased vigilance. There is a growing recognition that PTSD has adverse effects on health outcomes, including readmission rates, return to work rates, activities of daily living, feelings of rehabilitation, and opioid application.^[21–24]^ The need for adequate screening and early intervention for patients with PTSD symptoms is growing.

The purpose of this study was to determine the combined prevalence of PTSD in patients with acute trauma and to estimate the combined effects of related factors as well. The study will try to fill this gap by providing new evidence-based results to attract the attention of decision makers. The results of this study are expected to determine problems for future research.

## Author contributions

**Conceptualization:** Lang Wang.

**Data curation:** Furong Tang, Jianghong Tan, Xi Guo.

**Formal analysis:** Furong Tang.

**Funding acquisition:** Lang Wang.

**Project administration:** Lang Wang.

**Resources:** Xi Guo.

**Software:** Jianghong Tan, Xi Guo.

**Supervision:** Xi Guo, Jinlian Huang, Jinhua Yi.

**Validation:** Jinlian Huang, Jinhua Yi.

**Visualization:** Jianghong Tan, Jinlian Huang.

**Writing – original draft:** Lang Wang, Furong Tang, Jianghong Tan.

**Writing – review & editing:** Lang Wang, Furong Tang, Jianghong Tan.
